# WSES worldwide emergency general surgery formation and evaluation project

**DOI:** 10.1186/s13017-018-0174-5

**Published:** 2018-03-13

**Authors:** Federico Coccolini, Yoram Kluger, Luca Ansaloni, Ernest E. Moore, Raul Coimbra, Gustavo P. Fraga, Andrew Kirkpatrick, Andrew Peitzman, Ron Maier, Gianluca Baiocchi, Vanni Agnoletti, Emiliano Gamberini, Ari Leppaniemi, Rao Ivatury, Michael Sugrue, Massimo Sartelli, Salomone Di Saverio, Walt Biffl, Fausto Catena

**Affiliations:** 1 General, Emergency and Trauma Surgery Department, Bufalini Hospital, 47521 Cesena, Italy; 2 Division of General Surgery Rambam Health Care Campus Haifa, Haifa, Israel; 3 Trauma Surgery, Denver Health, Denver, CO USA; 4 Trauma Surgery, Riverside University Health System Medical Center, Riverside, CA USA; 5 Faculdade de Ciências Médicas (FCM)—Unicamp Campinas, Campinas, SP Brazil; 6 Department of Surgery, Foothills Medical Centre, Calgary, Canada; 7 Department of Surgery, Trauma and Surgical Services, University of Pittsburgh School of Medicine, Pittsburgh, USA; 8 Department of Surgery, Harborview Medical Centre, Seattle, USA; 9 General and Emergency Surgery, Civili University Hospital, Brescia, Italy; 10 ICU Department, Bufalini Hospital, Cesena, Italy; 11 Second Department of Surgery, Meilahti Hospital, Helsinki, Finland; 12 Virginia Commonwealth University, Richmond, VA USA; 13 General Surgery Department, Letterkenny Hospital, Letterkenny, Ireland; 14 Department of Surgery, Macerata Hospital, Macerata, Italy; 15 Trauma Surgery, Cambridge University Hospitals NHS Foundation Trust, Cambridge, UK; 16 Emergency and Trauma Surgery, Scripps Memorial Hospital, La Jolla, CA USA; 17 Emergency Surgery, Parma Hospital, Parma, Italy

**Keywords:** Emergency general surgery, Formation, Evaluation, Implementation, Open letter, Trauma, Benchmarks, Certification, Register, Certification

## Abstract

Optimal management of emergency surgical patients represents one of the major health challenges worldwide. Emergency general surgery (EGS) was identified as multidisciplinary surgery performed for traumatic and non-traumatic acute conditions during the same admission in the hospital. EGS represents the easiest viable way to provide affordable and high-quality level of care to emergency surgical and trauma patients. It may result from the association of different physicians with other specialties in a cooperative model. The World Society of Emergency Surgery (WSES) has been working on the EGS organization and implementation since its foundation believing in the need of common benchmarks for training and educational programs throughout the world. This is a plea in different languages to all World Prime Ministers and Presidents to support the creation in all nations of an organized hub-spoke system for emergency general surgery to improve standards of care and to save lives.

Optimal management of emergency surgical patients represents one of the major health challenges worldwide [1, 2]. Emergency surgical procedures may interfere with the daily planned surgical activity and therefore overwhelm the unprepared system. Many medical systems are not ready to deal with concurrent emergency and elective surgical procedures. Emergency general surgery (EGS) was identified in the 2000s as multidisciplinary surgery performed for traumatic and non-traumatic acute conditions during the same admission in the hospital [3]. EGS represents the easiest viable way to provide affordable and high-quality level of care to emergency surgical and trauma patients taking into account the pathophysiology, surgical and trauma emergencies, and critical care [4]. In several countries, it may result from the association of different physicians with other specialties in a cooperative model (i.e., emergency physicians, intensivists). In low-resource settings, these different providers may even be represented by the only general surgeon. The critically ill EGS patient requires prompt evaluation and, in many cases, early surgical intervention because of the uniqueness of the surgical acute conditions which are accompanied by high rates of complications and death [4]. The aforementioned landscape of EGS highlights the difficulty for the emergency general surgeon to plan and endorse appropriate management schemes to optimize timely treatment.

Shortage of trained emergency general surgeons, lack of dedicated teams, shortage of dedicated operating theaters, and delay in surgery as well as non-adherence to clinical guidelines may affect patient outcomes.

The last two decades have witnessed a slow but progressive improvement in the management of emergency general surgery cases. In many institutions, emergency general surgery services have become of critical importance.

Many governments have started a systematic effort in reorganizing EGS systems [5, 6]. They have begun to change the traditional paradigm of care and have renewed interest in the optimization of the processes and outcomes of care in EGS [6]. In fact, several countries and governments started to dedicate properly trained personnel in managing EGS patients within dedicated protocols and pathways.

Acute Care Surgery (ACS) was introduced at the beginning of 2000, as a well-defined concept. However, its practice paradigm has been in place for the past 50 years in the USA. The practice was developed in the large county hospitals in the USA in the late 1960s/early 1970s when trauma centers became recognized, and virtually all were housed in county hospitals. In these hospitals, the general surgeon providing trauma call did all emergent operations except for the central nervous system [7]. Even if widely recognized, it is not a stand-alone surgical specialty so far. In any case, data indicate that this concept has an impact on morbidity and mortality. In fact, it is an evolving concept encompassing three essential components: trauma, surgical critical care, and emergency general surgery with a formal training, certification, and ongoing practice of critical care, which has not yet found its proper position in the surgical panorama [8–12]. The American Association for Surgery for Trauma (AAST) defined a formative curriculum for acute care surgeons [13]. However, most institutions around the world are not prepared to offer such complete and articulated training program. In Europe, for example, an effort to define this subspecialty was attempted [11, 12] but currently there are no widespread set of minimum standards for ACS formation and specialization [10]. For the same reasons, some confusion exists in defining, outside the USA, what is the proper scope of practice of ACS and the specific providers. In fact, in most of the world, general surgeons, associated to other clinicians in a dedicated team to EGS patients, practice EGS by necessity and not based on specific training in the specialty. For these reasons, EGS is a progressive, more diffuse model. The system where ACS has been conceptualized and applied is different from most parts of the world. Most systems around the world have different political and economical organization. The EGS model is more flexible and, as a consequence, easily disseminated and reproducible.

Trauma disciplines have been developed and structured in several parts of the world. Many educational endeavors were put forward: ATLS® (Advanced Trauma Life Support), DSTC™ (Definitive Surgical Trauma Care), ATOM® (Advanced Trauma Operative Management), ASSET (Advanced Surgical Skills for Exposure in Trauma), FIAT (Full Immersion in Acute care surgery and Trauma), USET (Ultrasound in Emergency & Trauma), and ADMR (Advanced Disaster Medical Response) to improve outcomes and to standardize management. Guidelines were set forth and management algorithms established.

EGS should follow similar milestones. However, recent studies have demonstrated that the “one-size-fits-all” model of ACS does not work [14, 15]. Besides institutional and governmental differences in how health care delivered is organized, the practice of EGS across hospitals is mainly led by interested surgeons. In order to become widespread and effective, it should include scientific professional societies, stakeholders, and policymakers, especially in those situations at high risk of lacking access to high-quality emergency general surgical care [15]. The need for specific training, scientific knowledge based on high-level research, big data accrual, and the development of guidelines to stratify diseases requiring urgent surgical intervention is consolidating with the centers’ stratification in a hub-spoke model. Moreover, the necessity of critical care knowledge is becoming day by day more evident and mandatory.

Additionally, the development of common standards defining an EGS service is essential in order to reduce the legal burden on emergency general surgeons.

The World Society of Emergency Surgery (WSES) has been working on the EGS organization and implementation since its foundation. Several steps have been done but further improvements are mandatory to warrant an equal distribution of knowledge and resources. Moreover, the WSES strongly believes in the need of common benchmarks for training and educational programs throughout the world.

The acute care surgeon’s formation needs to be promoted together with the necessary recognition and outcomes evaluation and performance improvement. To reach such articulated aim, WSES recognized and initiated some key steps and the subsequent activities.

The key steps to build up this common program are:

1. Recognition
2. Mandatory priorities evaluation (taken into consideration the context: social, economic, political, religious, scientific, etc.)
3. Definition of the lowest common denominator for action criteria (guidelines including sections dedicated to low-resource settings)
4. Definition of training courses shared within the different contexts and containing the lowest common denominator action criteria
5. Well-defined quality improvement processes
6. Data accrual instrument set-up
7. Data analysis (indexing and stratification by the different contexts)
8. Result dissemination and dedicated restitution to the different centers to evaluate the necessities and to improve outcomes
9. Official certification of the different centers (with consequent eventual stratification)

*1, 2, 3 - Recognition*

*- Mandatory priorities evaluation (taken into consideration the context: social, economic, political, religious, scientific, etc.)*

*- Definition of the lowest common denominator for action criteria (guidelines including sections dedicated to the low-resource settings)*

In the last 10 years, the WSES has set-up and implemented the National Delegate Project with the aim to have a direct local qualified delegate in each country of the world reporting on the local situation and helping in refining the WSES guidelines, position papers, and consensus conferences according to each country’s needs. This project resulted in several EGS guidelines elaborated and published with a global perspective.

*4 Definition of training courses shared within the different contexts and containing the lowest common denominator action criteria*

Several courses have been organized and disseminated around the world to promote EGS formation (i.e., Management of Intra-abdominal infections Course “MIC course,” Emergency Abdominal Surgery Course “EASC,” Comprehensive Open Abdomen Management Course “COPAM course,” Intensive Care for Emergency General Surgeons “ICE course,” Base Evidences in Emergency General Surgery Course “BEES course,” Base Evidences in Trauma Surgery “BETS course,” Research In Surgery Course “RIS course,” Preparedness and Education of Acute care surgeons to mass Casualties Emergencies “PEACE course”). Moreover, all courses have been put together into a unique formative program the Full Immersion in Acute care surgery and Trauma “FIAT course”.

*5 Quality check instrument definition*

A restricted number of variables necessary to evaluate the EGS units/systems performance in both process and clinical outcomes have been established [16].

These key performance indicators (KPIs) take into account the:

Resources and Designation of Emergency Surgery Service

- Acute care unit structure
- Reception and triage
- Data systems, registry, and evaluation
- Rural emergency care and transfer
- Pediatric emergency general surgery care
- Geriatric emergency general surgery
- Interaction and connectivity within the health system
- Emergency room
- Laboratory
- Radiology
- Operating room
- Intensive care unit
- Gastroenterology
- Quality assurance and performance improvement and innovation
- Sepsis control
- Emergency room
- Intensive care
- Research in emergency general surgery
- Education in emergency general surgery
- Accreditation review and consultative program
- Patient-related outcome measures

*6, 7 - Data accrual instrument set-up*

*- Data analysis*

The WIRES project (WSES International Registry of Emergency General Surgery) has been set up to allow to all the EGS surgeons to register their activity and to obtain a worldwide register of surgical emergencies. This will give the opportunity to evaluate results on a macro-data basis and to give index allowing stratifying, evaluating, and improving the outcomes.

*8, 9 - Results diffusion and dedicated restitution to the different centers to evaluate the necessities and to improve outcomes;*

*- Official certification of the different centers (with consequent eventual stratification)*

Data analysis from the WIRES project are published in the *World Journal of Emergency Surgery* (WJES), an open access peer review journal, to spread the knowledge to everybody with no limitations due to the open access policy. The single-center/unit results can be discussed with the specific center/unit by international experts in order to help improve outcomes.

Lastly, this entire project will definitely lead to the release of an international certification linked to the WSES Official World Certification Process (WOWcp) project. This certification warrants the outcomes of the centers/unit to be in the standard of the best practice of EGS.

Emergency general surgery treats time-sensitive diseases: this is a plea in different languages to all World Prime Ministers and Presidents to support the creation in all nations of an organized hub-spoke system for emergency general surgery to improve standards of care and to save lives.

## Abbreviations

AAST: Association for Surgery for Trauma
ACS: Acute Care Surgery
ADMR: Advanced Disaster Medical Response
ASSET: Advanced Surgical Skills for Exposure in Trauma
ATLS®: Advanced Trauma Life Support
ATOM®: Advanced Trauma Operative Management
BEES: Base Evidences in Emergency General Surgery Course
BETS: Base Evidences in Trauma Surgery
COPAM: Comprehensive Open Abdomen Management Course
DSTC™: Definitive Surgical Trauma Care
EASC: Emergency Abdominal Surgery Course
EGS: Emergency general surgery
FIAT: Full Immersion in Acute care surgery and Trauma
ICE: Intensive Care for Emergency General Surgeons
MIC: Management of Intra-abdominal infections Course
PEACE: Preparedness and Education of Acute care surgeons to mass Casualties Emergencies
RIS: Research In Surgery Course
USET: Ultrasound in Emergency & Trauma
WIRES: WSES International Registry of Emergency General Surgery
WOWcp: WSES Official World Certification Process
WSES: World Society of Emergency Surgery
